# Supplementary material for: Diguanylate Cyclase (DGC) Implicated in the Synthesis of Multiple Bacteriocins via the Flagellar-Type III Secretion System Produced by Pectobacterium carotovorum subsp. carotovorum
Source: Int J Mol Sci. 2022 May 18;23(10):5649. doi: 10.3390/ijms23105649 (PMC9144310; doi:10.3390/ijms23105649)
Supplement: Supplementary file 1 [file ijms-23-05649-s001.zip › ijms-1708715-supplementary.pdf]

A

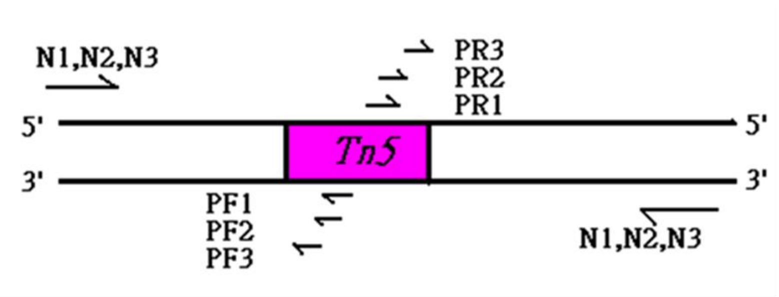

B

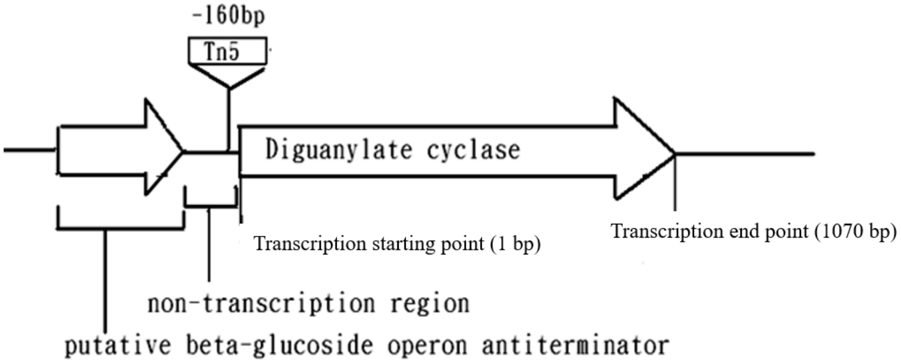

C

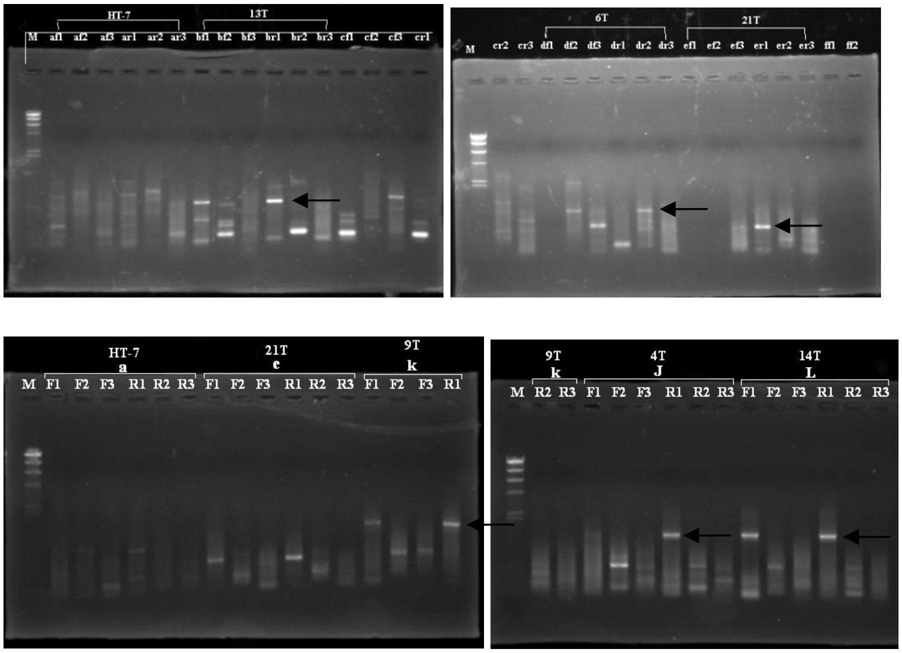

Figure S1. The Thermal Asymmetric Interlaced PCR (TAIL-PCR) and the results from the process. A. Schematic diagram of the TAIL-PCR process. Three contiguous specific primers from the Tn5 insert end sequence are used to amplify unknown nucleotides, with the same arbitrary primer used in each reaction.; B. The Tn5 insertion map of Diguanylate Cyclase gene. Tn5 has been inserted into the Diguanylate Cyclase gene, which has a total length of 1070bp and Tn5 has been put into -160bp.; (c) Results from the TAIL-PCR.
